# A chemoproteomic method for identifying cellular targets of covalent kinase inhibitors

**DOI:** 10.18632/genesandcancer.106

**Published:** 2016-05

**Authors:** Ying-Chu Chen, Chao Zhang

**Affiliations:** ^1^ Department of Chemistry and Loker Hydrocarbon Research Institute, University of Southern California, Los Angeles, CA, USA

**Keywords:** protein kinases, covalent inhibitors, target identification

## Abstract

Protein kinases are attractive drug targets for numerous human diseases including cancers, diabetes and neurodegeneration. A number of kinase inhibitors that covalently target a cysteine residue in their target kinases have recently entered use in the cancer clinic. Despite the advantages of covalent kinases inhibitors, their inherent reactivity can lead to non-specific binding to other cellular proteins and cause off- target effects in cells. It is thus essential to determine the identity of these off targets in order to fully account for the phenotype and to improve the selectivity and efficacy of covalent inhibitors. Herein we present a detailed protocol for a chemoproteomic method to enrich and identify cellular targets of covalent kinase inhibitors.

## INTRODUCTION

Protein kinases are a large family of enzymes that transfer the γ-phosphate group of ATP to the tyrosine, serine or threonine residues of substrate proteins thus modulating numerous biological processes in eukaryotes. There are approximately 530 protein kinases in human, which constitute about 1.7% of all human genes [1]. Kinases play important roles in signal transduction and thus regulate a variety of cellular processes including metabolism, transcription, cell cycle progression, cytoskeletal rearrangement, cell movement, apoptosis and differentiation [2, [3]. Mutations and dysregulation of protein kinases have been implicated in numerous human diseases including cancers, diabetes and neurodegeneration [4, [5]. Frequent occurrence of the disease-causing mutations in protein kinases make them attractive targets for therapeutic discovery.

Numerous small molecules have been tested for inhibition against protein kinases and evaluated as targeted cancer therapies. Approximately 30 kinase inhibitors have been approved by the FDA for treating various types of cancer in the clinic [6]. Imatinib (Gleevec), a small-molecule inhibitor of the oncogenic fusion kinase BCR-ABL, was first approved by the FDA in 2001 for the treatment of chronic myeloid leukemia (CML) [7]. Subsequently, numerous other kinase inhibitors, such as gefitinib, erlotinib, sorafenib, sunitinib, lapatinib, dasatinib, crizotinib, and vemurafenib, have been approved by the FDA for the treatment of a variety of cancers including non-small cell lung carcinoma, breast cancer, hepatocellular carcinoma, renal cell carcinoma and melanoma [6].

The majority of clinically approved kinase inhibitors rely on non-covalent forces such as hydrogen bonds, ionic bonds and van der waals interactions to bind to the kinase active site [6]. A small number of kinase inhibitors can form covalent interactions with the sulfurhydryl group of cysteine in protein kinases [8]. Such covalent interactions provide a number of advantages including high selectivity and potency against the target of interest, as well as prolonged and tunable pharmacodynamics [9, [10]. Highly specific inhibitors have been identified for individual kinases by covalently targeting non-conserved, rare cysteine in or near the kinase active site [8-13].

**Figure 1:**
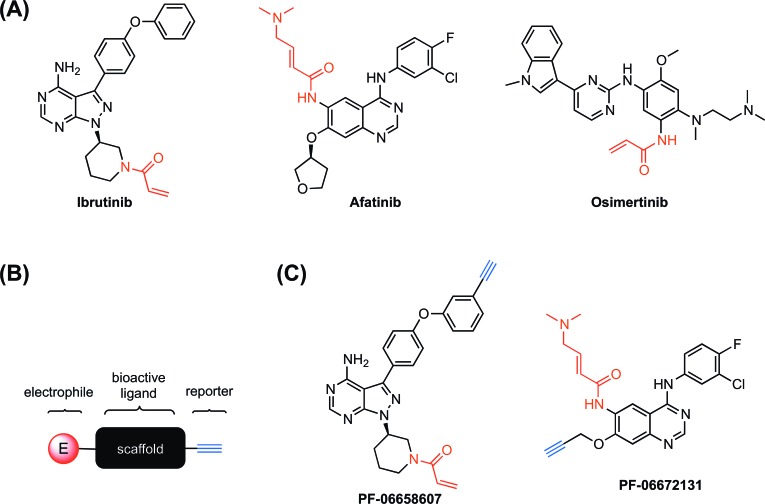
Examples of covalent kinase inhibitors and their alkyne derivatives (A) Covalent kinase inhibitors that are currently used in the clinic. (B) A schematic representation of covalent probe composition that contains a scaffold (black) for kinase binding, an electrophile (red) for covalent labeling, and a terminal alkyne group (blue) as a “clickable” reporter. (C) Derivatives of covalent kinase inhibitors that contain a terminal alkyne group as a reporter.

A number of covalent kinase inhibitors have entered clinic use. Afatinib, a covalent inhibitor of the epidermal growth factor receptors (EGFR), was approved by the FDA for the treatment of EGFR-driven non-small cell lung carcinoma (NSCLC) in 2013 (Figure 1A). EGFR receptor tyrosine kinase (RTK) subfamily includes four members in mammals: EGFR (ErbB1), ErbB2, ErbB3, and ErbB4, which play essential roles in cell proliferation, survival and differentiation [14]. Mutations and overexpression of EGFR are observed in various cancer cell types [15, [16]. In addition to wild-type EGFR, afatinib irreversibly binds and inhibits ErbB2, ErbB4, and certain EGFR mutants, including those caused by EGFR exon 19 deletion mutations or exon 21 (L858R) substitution mutations, as well as EGFR T790M gatekeeper mutation. The inhibition of these RTKs can result in the inhibition of tumor growth and angiogenesis in tumor cells overexpressing these RTKs. Afatinib carries an electrophilic acrylamide group for targeting Cys797 near the end of the EGFR kinase hinge region, which was confirmed by co-crystal structure [13]. Shortly after the FDA approval of afatinib, ibrutinib, a covalent inhibitor of Bruton's tyrosine kinase (BTK), was first approved by the FDA in 2013 for the treatment of mantle cell lymphoma (MCL) and later approved for the treatment of chronic lymphocytic leukemia (CLL) and Waldenström macroglobulinemia (Figure 1A) [17]. A member of the TEC family of non-receptor tyrosine kinases, BTK is a key regulator for B cell receptor (BCR) signaling and was found overexpressed in a number of B-cell malignancies [12]. Ibrutinib contains an acrylamide group that forms covalent interaction with Cys481 in BTK (at a homologous position to Cys797 in EGFR) and inhibits BTK kinase activity thus preventing BCR signaling [17].

Inspired by the clinical success of afatinib and ibrutinib, there are currently extensive efforts focusing on the development of irreversible kinase inhibitors [9, [18]. For example, osimertinib, a selective covalent inhibitor for the drug-resistant mutant (T790M) of EGFR, has been recently approved by the FDA for treating metastatic NSCLC (Figure 1A) [11]. As compared to previous FDA-approved EGFR inhibitors that also inhibit wild- type EGFR, osimertinib demonstrated great selectivity for EGFR T970M that is only harbored in tumors thus reducing toxicity on normal cells [19].

Despite the advantages of covalent kinases inhibitors described above, the inherent reactivity of covalent inhibitors can lead to non-specific binding to other cellular proteins and cause off-target effects in cells [10]. The covalent modification of off-targets may complicate the analysis of signaling transduction in cells as well as increase the risk of hapten formation (triggering an immune response to the adducted protein) and could lead to high cytotoxicity due to the sustained off-target engagement [20, [21]. It is thus essential to determine the identity of these off targets in order to fully account for the phenotype and to improve the selectivity and efficacy of covalent inhibitors [22].

**Figure 2:**
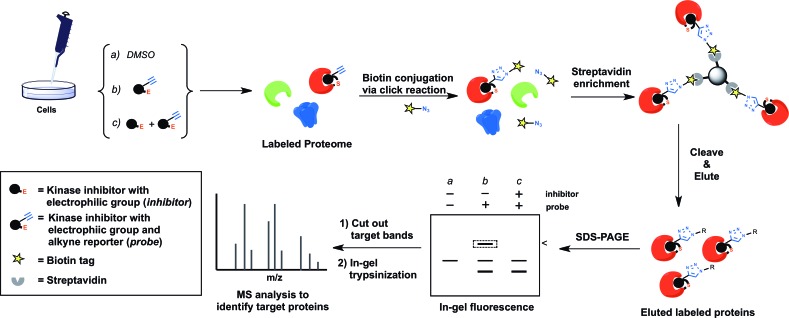
A schematic representation of the procedure to enrich and identify cellular targets of covalent kinase inhibitors

Target identification of covalent kinase inhibitors can be achieved by using a method that involves selective pull-down of target proteins and mass spectrometry for protein identification [23]. First, the covalent kinase inhibitor is derivatized with a terminal alkyne group to generate a probe compound (Figure 1B). Cells will be treated with the probe resulting in all cellular targets covalently modified with an alkyne tag (Figure 2). After cell lysis, the lysate is subjected to the copper(I)- catalyzed alkyne-azide cycloaddition (CuAAC) click chemistry to conjugate those target proteins with a biotin tag. The biotin-tagged proteins can then be pulled down on streptavidin resin before the target proteins is selectively eluted by cleaving an azo-linker in the tag with sodium dithionite (Figure 2). The proteins enriched in the eluent can be visualized by SDS-PAGE gel analysis and identified by mass spectrometry analysis. The confidence of target relevance can be evaluated by including negative control of no probe treatment or pretreatment of the original covalent kinase inhibitor to compete off probe labeling (Figure 2).

Cells are first treated with DMSO, the probe alone, or the inhibitor followed by the probe before being lysed. Probe-labeled proteins are conjugated with biotin via CuAAC click reaction, pulled down with streptavidin, eluted with sodium dithionite, and then resolved by SDS-PAGE. Protein bands are cut out, digested by trypsin, and finally subjected to MS analysis for target identification.

Cravatt and co-workers have applied a similar method to determine the protein targets of two clinically used covalent kinase inhibitor drugs, afatinib and ibrutinib, in the entire human proteome [24]. Notably, they discovered both kinases and non-kinase proteins among the identified cellular targets of both covalent drugs (Figure 1C).

Herein we present a detailed protocol for this chemoproteomic method. It can serve as a general method for enriching and identifying targets of not only covalent kinase inhibitors but also covalent probes targeting non- kinase proteins.

## REAGENTS

- DMSO - dimethylsulfoxide (EMD cat. no. MX1458-6)
- DPBS - Dulbecco's phosphate-buffered saline (Corning, cat. no. 55-031-PC)
- Protease inhibitor cocktail (Roche, cat. no. 11836170001)
- PMSF - phenylmethylsulfonyl fluoride (Amresco, cat. no. 0754)
- NP40 - Nonidet P40 substitute (Sigma-Aldrich, cat. no. 74385) NaCl (EMD OmniPur, cat. no. 7710)
- HEPES - 4-(2-hydroxyethyl)-1- piperazineethanesulfonic acid (EMD OmniPur, cat. no. 5320)
- Pierce BCA protein assay kit (Thermo scientific, cat. no. 23225)
- Biotin-azo-azide (Click Chemistry Tools, cat. no. 1041)
- TCEP - tris(2-carboxyethyl) phosphine hydrochloride (Thermo Scientific, cat. no. 20490)
- TBTA - tris-[(1-benzyl-1H-1,2,3-triazol-4-yl) methyl]amine (TCI, cat. no. T2993)
- Copper (II) sulfate pentahydrate (Sigma- Aldrich, cat. no. 209198)
- 2X Laemmli sample buffer (Bio-Rad, cat. no. 1610737)
- MeOH - methanol (EMD cat. no. MXD475-1)
- Urea (EMD Calbiochem, cat. no. 666122)
- Thiourea (Amresco, cat. no. M226)
- DTT - dithiothreitol (EMD OmniPur, cat. no. 3860)
- Iodoacetamide (Sigma-Aldrich, cat. no. 16125)
- Streptavidin Agrose (Thermo Scientific, cat. no. 20349)
- PBS - phosphate buffered saline, pH 7.4 (Sigma-Aldrich, cat. no. P5368)
- SDS - sodium dodecyl sulfate (Amresco, cat. no. 0227)
- Sodium dithionite (Sigma-Aldrich, cat. no. 157953)
- Tris HCl - tris(hydroxymethyl) aminomethane (EMD OmniPur, cat. no. 9310)
- Bromophenol blue (EMD cat. no. BX1410)
- Glycerol (EMD cat. no. GX0185-5)
- β-mercaptoethanol (Sigma-Aldrich, cat. no. M3148)
- Colloidal blue staining kit (Invitrogen, cat. no. LC6025)

## REAGENT PREPARATION

- 1% NP40 buffer (1% NP40, 150 mM NaCl, 50 mM Hepes pH 7.4)
- PMSF solution (125 mM stock in H_2_O, prepare fresh and vortex prior to use)
- Biotin-azo-azide stock solution (5 mM stock solution in DMSO)
- TCEP solution (50 mM stock solution in H_2_O, prepare fresh prior to use)
- TBTA stock solution (10 mM stock solution in DMSO)
- CuSO_4_ solution (50 mM stock solution in H_2_O, concentration using the BCA assay. prepare fresh prior to use)
- Resuspension buffer (6 M urea, 2 M thiourea, 10 mM Hepes, pH 8.0, prepare fresh prior to use)
- DTT solution (100 mM in H_2_O, prepare fresh prior to use)
- Iodoacetamide solution (550 mM in H_2_O, prepare fresh prior to use)
- 1% SDS in PBS (1% (wt/vol) SDS in PBS buffer)
- Sodium dithionite buffer (50 mM Na_2_S_2_O_4_and 1% SDS in PBS, prepare fresh prior to use)
- 4% SDS buffer (4% SDS, 150 mM NaCl, and 50 mM Hepes, pH 7.4)
- 2X SDS-free loading buffer (0.2% bromophenol blue, 20% glycerol, 5% β-mercaptoethanol, and 100 mM Tris, pH 6.8)

## PROCEDURE

### Live cell proteome labeling

1. Seed cells in 15 cm petri dishes and grow to ∼90% confluency in appropriate medium. Three 15 cm petri dishes are typically required for each condition, e.g. probe treatment or control.
2. Replenish with 20 mL of fresh medium and add 20 uL of probe solution (1,000x stock in DMSO). Gently swirl the plate to mix. Same volume of DMSO is added to the control cells.
3. After incubation at 37°C for 1 h, aspirate medium, wash cells twice with DPBS, and harvest cells in 5 mL DPBS using a cell scraper. Combine cell suspension from three plates and transfer to a 15 mL centrifuge tube.
4. Centrifuge cells at 2,000 × g and 4°C for 2 min. Carefully aspirate supernatant without disturbing the pellet. Proceed to cell lysis and biotin-azide conjugation.

### Cell lysis and CuAAC conjugation

1. Dissolve protease inhibitor cocktail (25 mg, Roche) in 500 μL of 1% NP40 buffer by bath sonication. Add 30 μL of 125 mM PMSF solution to prepare the lysis buffer. Resuspend the cell pellet in 530 uL of the above ice-cold lysis buffer. The resulting cell lysate is diluted with 800 μL of 1% NP40 buffer.
2. Sonicate the cell lysate in bath sonicator for 5 min and incubate on ice for another 30 min before centrifugation at 20,000 × g at 4°C for 10 min (or 10,000 × g and 4°C for 20 min). Take the supernatant and determine protein concentration using the BCA assay.
3. Each sample is diluted to a final volume of 9.4 mL that contains 10 mg of total protein. Add the following reagents sequentially to reach a final volume of 10 mL and gently vortex the sample after each addition.
4. 100 μL of 5 mM biotin-azo-azide (50 μM final concentration)
5. 200 μL of 50 mM TCEP (1 mM final
6. 100 μL of 10 mM TBTA (100 μM final concentration)
7. 200 μL 50 mM CuSO_4_ (1 mM final concentration)
8. Incubate at room temperature (RT) for 1 h in the dark. Add 40 mL of methanol to the reaction mixture. Keep at −20°C overnight. Proceed to target protein enrichment.

### Enrichment of probe-labeled proteins

1. Pellet proteins by centrifugation at 5,200 × g and 0°C for 30 min before decanting the supernatant. Wash the residue by resuspending the pellet with 40 mL ice-cold methanol, centrifuging at 5,200 × g and 0°C for 30 min, and removing supernatant. Repeat methanol wash twice more.
2. Air-dry pellets by placing the tube upside down with an angle of 45 degree for 30 min. Resuspend protein pellet with bath sonication in 4 mL of resuspension buffer to achieve a final concentration of 1.25 mg/mL (assuming 50% protein loss).
3. Add 40 uL of DTT solution and incubate for 40 min at RT. Subsequently add 40 uL of iodoacetamide solution and incubate for 30 min in the dark at RT.
4. Pre-Wash streptavidin beads (250 μL) with PBS (2 × 1 mL) and resuspension buffer (1 × 1 mL). Centrifuge at 2,000 × g for 3 min at RT to collect the beads. Remove supernatant carefully. Add resuspended proteins to streptavidin beads in 15 mL centrifuge tube and incubate on a rotator for 2 h at RT.
5. Collect beads by centrifugation at 2,000 × g for 3 min at RT. Wash beads sequentially with resuspension buffer (2 × 10 mL), PBS (2 × 10 mL), and 1% SDS in PBS (2 × 10 mL). Transfer beads to a 2 mL dolphin-nose tube using wide- bore pipette tips.
6. Add 200 μL sodium dithionite buffer to beads and incubate at RT for 30 min with gentle rocking. Collect supernatant by centrifugation at 2,000 × g for 3 min at RT. Repeat the elution once more and combine supernatant.
7. Add 1.6 mL ice-cold MeOH to the combined supernatant and incubate at −20°C overnight. Centrifuge at 10,000 × g for 10 min at 4°C to pellet protein before decanting supernatant. Place tube up side down with an angle of 45 degree to air-dry the pellet. Proceed to SDS- PAGE gel analysis.

### SDS-PAGE gel analysis

1. Resuspend protein pellet in 15 μL of 4% SDS buffer, bath sonicate briefly, and then add 15 μL of 2X SDS-free loading buffer.
2. Boil the sample at 98°C for 5 min and centrifuge at 10,000 × g for 3 min at RT before loading all 30 μL of the sample into SDS-PAGE gel. 20 μg of whole cell lysate can be used as input for comparison.
3. The gel is stained with Colloidal Blue for 3 h and then destained with water overnight according to manufacture's manual.
4. Cut out the desired gel slices and send out for mass spectrometry analysis to identify the proteins.
